# Opioid substitution treatment and heroin dependent adolescents: reductions in heroin use and treatment retention over twelve months

**DOI:** 10.1186/s12887-018-1137-4

**Published:** 2018-05-04

**Authors:** Bobby P. Smyth, Khalifa Elmusharaf, Walter Cullen

**Affiliations:** 1 Department of Public Health and Primary Care, Trinity College Dublin, Dublin 2, Ireland; 2 Graduate Entry Medical School, University of Limerick, Limerick, Ireland; 3 National Drug Treatment Centre, 30/31 Pearse St, Dublin 2, Ireland; 4 Academic General Practice, School of Medicine, University College Dublin, Belfield, Dublin 4 Ireland; 5 HSE Addiction Service, Bridge House, Cherry Orchard Hospital, Dublin 10, Ireland

**Keywords:** Adolescents, Heroin dependence, Methadone treatment, Buprenorphine treatment, Treatment adherence

## Abstract

**Background:**

Opioid dependence is a major health concern across the world and does also occur in adolescents. While opioid substitution treatment (OST) has been thoroughly evaluated in adult populations, very few studies have examined its use in adolescents. There are concerns that OST is underutilised in adolescents with heroin dependence. We sought to measure changes in drug use among adolescents receiving OST and also to examine treatment attrition during the first 12 months of this treatment.

**Methods:**

We included all heroin dependent patients aged under 18.5 years commencing OST at one outpatient multidisciplinary adolescent addiction treatment service in Dublin, Ireland. Psycho-social needs were also addressed during treatment. Drug use was monitored by twice weekly urine drugs screens (UDS). Change in the proportion of UDS negative for heroin was examined using the Wilcoxon signed rank test. Attrition was explored via a Cox Regression multivariate analysis.

**Results:**

OST was commenced by 120 patients (51% female and mean age 17.3 years). Among the 39 patients who persisted with OST until month 12, heroin abstinence was 21% (95% confidence interval [CI] = 9–36%) at month three and it was 46% (95% CI = 30–63%) at month 12. Heroin use declined significantly from baseline to month three (*p* < 0.001) and from month three to month 12 (*p* = 0.01). Use of other drugs did not change significantly. People using cocaine during month 12 were more likely to be also using heroin (*p* = 0.02). Unplanned exit occurred in 25% patients by 120 days. The independent predictors of attrition were having children, single parent family of origin, not being in an intimate relationship with another heroin user and evidence of cocaine use just before treatment entry.

**Conclusions:**

We found that heroin dependent adolescent patients achieved significant reductions in heroin use within three months of starting OST and this improved further after a year of treatment, about half being heroin abstinent at that stage. Patient drop out from treatment remains a challenge, as it is in adults. Cocaine use before and during treatment may be a negative prognostic factor.

**Electronic supplementary material:**

The online version of this article (10.1186/s12887-018-1137-4) contains supplementary material, which is available to authorized users.

## Background

There is global ongoing concern regarding opioid dependence. While this is primarily focused on non-prescribed use of pharmaceutical opioids in USA currently, heroin has been the main concern elsewhere, including Ireland [1, 2]. The prevalence of heroin dependence tends to fluctuate over time and is much more common in adults than adolescents [1, 3]. In Dublin, heroin use first emerged as a concern among adolescents in the 1990s, in the midst of a dramatic increase in heroin dependence among adults [3, 4].

Opioid substitution treatment (OST) is now the main first line treatment intervention for heroin dependence among adults [5]. The main medications used are methadone, a full agonist or buprenorphine, a partial agonist. There are legislative and administrative obstacles in many jurisdictions to provision of OST to adolescents [1]. The limited evidence base for this treatment in this younger age range is also an issue which may be contributing to wariness by clinicians to prescribe [2, 6]. A recent study in USA found that just 2.4% of adolescents attending treatment for heroin use received medication [1].

There are no clinical trials examining outcome of OST beyond three months in people aged under 18 years old [6]. There are a small number of ‘open label’ longer term studies. The largest study has involved just 153 patients and was conducted over 40 years ago [7]. However, it provided no information on cessation of heroin use among those who remained on methadone treatment (MT), and the dose of methadone was limited to just 20mgs so its implications for modern practice are very unclear. Although the primary goal of OST is to assist the patient in reducing their heroin use, the other adolescent studies also fail to provide any firm information on this issue. The absence of scientific information on the rates of ongoing heroin use by adolescents on OST acts as an obstacle to provision of this treatment [2].

The DARP study of adolescents in USA in the 1970s did report a progressive decline in heroin use over the first year of MT but it did not provide any specific information on the actual rates of heroin use during treatment [8]. More recently, the NIDA multisite buprenorphine/naloxone treatment (BT) trial examined treatment of youth over a three month period, although only 12 participants were under eighteen years-old. At week eight, 77% of those retained on BT provided an opiate negative urine drug screen [9]. Abstinence from heroin at the end of that trial was associated with greater baseline medical and psychiatric problems, a history of recent injecting and evidence of early cessation of heroin use during BT [10].

Adolescents entering addiction treatment typically present with polysubstance use and this is also the case for those with primary dependence upon heroin [11–13]. An adolescent’s motivation to address use of one substance such as heroin may not generalise to other substances [13]. Although there is evidence that adults on OST can also achieve significant reductions in use of other drugs, there is no such evidence for OST programs for adolescents [14]. The DARP study from the USA in the 1970s reported evidence of increased use of cannabis and alcohol among youth on OST at follow up [8].

Drop out from OST has been examined by a small number of studies in this age range. Studies of MT indicate that 25% of patients drop out within approximately four months [7, 8, 15]. Bell & Mutch found that the drop out from BT was significantly greater than from MT [16]. Other studies of BT in this age range also indicate frequent early drop out from treatment, with typically a quarter of patients exiting within 4 to 8 weeks [17–19].

Given this paucity of outcome studies of OST in adolescent heroin dependence, we sought to address a number of questions which are of importance to clinicians and patients in order to make informed decisions. What proportion of adolescents ceases heroin use while on OST? Does abstinence increase incrementally or simply stabilise beyond three months on OST? What is the rate of drop out? Are there any baseline or treatment characteristics which are associated with either abstinence or drop out?

## Methods

### Setting, assessment and treatment

This study was based at the Young Persons’ Programme (YPP) within the National Drug Treatment Centre (NDTC) in Dublin, Ireland. The approach to assessment and treatment of heroin dependent adolescents at this service is summarised below but has been described in detail elsewhere [20, 21]. Although published a number of years after the establishment of the YPP, the approach to treatment is consistent with the UK guidance on pharmacological management of substance dependence among young people [22].

Assessment typically involved three clinic visits to meet different members of the multi-disciplinary team, a supervised urine drug screen (UDS) being obtained at each visit. In addition to assessing for heroin dependence using ICD-10 criteria, co-existing psychological, developmental, social and physical needs were identified.

The main pillars of treatment involved OST (methadone or buprenorphine), counselling and in some cases, family therapy. Induction onto a stable dose of methadone typically occurred over a period of about two weeks while buprenorphine induction took 2–3 days [23]. If patients continued to use heroin or resumed use after a period of abstinence, an increase in medication was considered. Use of other substances was also addressed during treatment. Where the initial opioid agonist medication was associated with a poor treatment response, the patient was offered the option of switching to the alternative.

Patients provided two supervised UDS per week for on-site drug toxicology testing during treatment. These samples were screened for opiates, EDDP (a methadone metabolite), cocaine, benzodiazepines and alcohol. They were intermittently screened for amphetamines and cannabis. The provision of ‘take-away’ doses of medication was utilised as a contingent reinforcer of drug abstinence, as evidenced by urine toxicology.

Patients were also provided with treatment of co-morbid medical or psychiatric problems [21]. They were actively supported in addressing co-existing housing, vocational and criminal justice related needs as part of their care plan.

The overarching goal of the service was to reduce the harm which young people were experiencing related to their drug use. It held an aspiration, but not an insistence, that patients progress towards complete abstinence via gradual OST dose reduction and ultimate cessation of this medication. This ultimately occurs in about one in every four cases [20]. Where dose tapering occurred, it was generally conducted slowly on an outpatient basis over a period of about three months, negotiated with the individual patient.

### Patients

The inclusion criteria were:- heroin dependence, aged under 18.5 years and commenced OST. The cohort under study included patients who commenced treatment between May 2000 and June 2016. Patients with a primary diagnosis of dependence on prescription opioids were excluded, as such patients have been rarely encountered in Ireland.

### Data collection

The study was approved by the Research Ethics Committee of the NDTC. Baseline descriptive characteristics were obtained from the patients’ initial structured assessment, this being adapted from the Maudsley Addiction Profile [24]. We recorded information on patient treatment participation at three, six and twelve months. If a patient was not attending the service on these dates, we noted the date of and reason for exit.

Those who were referred to and commenced on another OST program were categorised as “transfers for ongoing treatment”. This typically involved transfer to an adult OST program, this option being available to patients after their 18th birthday. However, most opted to persist with treatment on the YPP beyond this age [20]. Patients were categorised as exiting via “dose taper” if they adhered to the prescribed dose reduction regime and showed urinalysis evidence of heroin abstinence at discharge from the treatment program. Patients who ended treatment because they were incarcerated were categorised as exiting due to “prison”. Finally, patients who simply stopped attending, relocated without arranging alternative treatment or left treatment prematurely against medical advice were all categorised as being “drop outs”. Patients were generally not deemed to have dropped out of their index treatment episode unless that had failed to attend for four weeks. If they were deemed a drop out, then the date of last attendance for medication was recorded as the date of discharge. Although many patients re-entered treatment after dropping out or following a relapse to heroin use after a successfully completed dose taper, only their first or index treatment episode was included for the purposes of this study. The pattern of transitions out of and back into treatment have been described in a separate study [20]. If a patient switched from BT to MT or vice versa, but continued in treatment, this was not viewed as a treatment exit.

To examine changes in drug use, five periods during the first year of treatment contact were examined for each patient. The results of all urine drug screens (UDS) provided by each patient during each period was collated. The periods in question were (a) pre treatment assessment phase which was typically 7 to 10 days in duration and involved provision of 2–4 UDS, (b) induction phase which comprised the remaining days in that first month of patient contact after commencing OST, (c) third entire month of treatment, (d) sixth entire month of treatment and (e) twelfth entire month of treatment. During phases (b) to (e), patients were typically providing two UDS per week. For each patient, a dichotomous outcome variable to indicate abstinence/use was created for each of the phases they were being treated (a) to (e). They were deemed to be using heroin if any one UDS sample tested positive for opiates during that phase. If all UDS were opiate negative, they were deemed heroin abstinent. Similarly, a single positive for cocaine in a month resulted in that month being deemed one of cocaine use.

### Statistics

In order to conduct an exploratory analysis, we used Pearson chi square test to examine association between categorical covariates and dichotomous outcome variables of interest, except in instances where a predicted cell value was less than five, where Fisher’s Exact test was utilised. Odds ratios and their 95% confidence intervals were calculated to indicate the direction and magnitude of associations. Continuous variables were converted into categorical variables, by choosing the median value as the point of split, apart from age where we used 18th birthday as the cut-off.

When reporting the proportion of patients who were heroin abstinent, we also calculated the 95% confidence intervals of those rates using the exact confidence limits for binomial proportions. McNemar’s paired proportions test with continuity correction was employed to determine whether OST was associated with changes in the binary category of drug use. We sought to confirm that heroin abstinence in month three was greater than baseline, and that abstinence at months six and twelve were greater than month three. We also examined changes in the proportion of samples which tested negative for heroin using the related samples Wilcoxon signed rank test. For each period, we calculated this proportion by dividing the number of negative tests by the total number of UDS obtained. All of these analyses were confined to the subgroup of patients who persisted with OST for a full year.

To examine drop out and factors associated with same, the Kaplan-Meier test was conducted. The event of interest was unplanned exit from treatment via drop out or imprisonment. The time to treatment exit, in days, was recorded for each patient who left their index treatment episode during the first year, whether by unplanned exit or via planned exit (following completion of dose taper or via transfer to another OST service). For patients who persisted with OST, the number of days entered was 365 days. The Log Rank was used to test for the equality of the survival distributions within each covariate. To facilitate interpretation of these differences, the estimated number of days to drop out by 25% is reported. In order to explore independent predictors of unplanned exit, we then conducted a multivariate analysis using Cox Regression. Covariates were selected for entry into the final equation using the forward and backward selection technique. All analyses were conducted using IBM SPSS Statistics, version 21.

## Results

### Description of participants

There were 120 eligible patients who commenced OST. One patient with primary methadone dependence was excluded and there were no presentations with primary prescription opioid dependence. The patient characteristics are provided in Table 1. The mean age was 17.3 years (range 14–18 years). There were 13 (11%) who had not yet reached their 16th birthday. Eighty-eight per cent were using heroin daily at treatment entry. Thirty-nine patients persisted with treatment to month 12 and their profile is also outlined in Table 1. Twenty-nine (24%) left following completion of dose taper and nine (8%) were transferred elsewhere for ongoing OST. There were 43 unplanned treatment exits, with 36 (30%) dropping out and 7 (6%) going to prison. There were no deaths during treatment.

**Table 1:** Characteristics of 120 adolescents commencing opioid substitution treatment, and subgroup who persisted in treatment until month 12

	Total Group	Missing data	In treatment until month 12			
	*N* (%)^c^		*N* (%)^d^	OR	95% CI OR	*P* value
Number in Treatment	120		39 (33)			
Socio-demographic characteristics						
Female	61 (51)		25 (41)	2.0	(0.9–4.4)	0.07
Aged under 18.0 years	104 (87)		35 (34)	1.5	(0.5–5.1)	0.49
Left school under 15 years	51 (46)	8	18 (35)	1.0	(0.5–2.3)	0.92
Not in employment, education or training	81 (70)	4	29 (36)	1.7	(0.7–4.1)	0.24
Two parent family support	59 (50)	1	27 (46)	3.8	(1.6–8.6)	0.001
Has a child	6 (5)		0 (0)	n/a		0.18^a^
Has been in care	38 (32)	2	10 (26)	0.7	(0.3–1.6)	0.35
Sibling Heroin Use	45 (39)	4	17 (38)	1.4	(0.6–3.0)	0.45
Parental heroin Use	25 (22)	4	6 (24)	0.6	(0.2–1.5)	0.25
Partner uses heroin	47 (39)	1	18 (38)	1.5	(0.7–3.3)	0.30
Homeless or hostel in past month	34 (28)		11 (32)	0.9	(0.4–2.2)	0.89
Previous criminal convictions	46 (41)	8	14 (30)	0.9	(0.4–2.0)	0.80
Ever incarcerated	31 (27)	6	8 (26)	0.8	(0.3–1.9)	0.55
Psychiatric History						
Ever assessed by a psychiatrist	62 (53)	2	22 (36)	1.4	(0.6–2.9)	0.44
Past Inpatient psychiatric admission	10 (9)	4	6 (60)	3.2	(0.8–12)	0.09^a^
Past DSH	36 (31)	5	12 (33)	1.1	(0.5–2.5)	0.86
Substance Use						
Lifetime Drug Use						
Non-prescribed benzodiazepines	107 (90)	1	36 (34)	1.0	(0.3–3.6)	1.0^a^
Non-prescribed methadone	90 (78)	5	30 (33)	0.8	(0.3–1.9)	0.54
Cocaine use	76 (68)	8	30 (40)	2.7	(1.1–7.0)	0.04
Injected	53 (45)	2	17 (32)	1.0	(0.5–2.2)	0.99
Commenced heroin under 15 years of age	43 (36)	2	20 (47)	2.6	(1.2–5.7)	0.02
Regular heroin use for more than 12 months	73 (64)	6	26 (36)	1.2	(0.5–2.7)	0.67
Past Month Drug Use						
Non-prescribed benzodiazepines	71 (60)	1	26 (37)	1.4	(0.6–3.1)	0.40
Non-prescribed methadone	71 (60)	1	27 (38)	1.7	(0.7–3.7)	0.22
Cocaine	31 (26)	2	10 (32)	1.0	(0.4–2.3)	0.91
Cannabis	80 (73)	11	27 (34)	1.3	(0.5–3.4)	0.54
Amphetamine	9 (9)	19	4 (44)	1.9	(0.5–7.7)	0.45^a^
Alcohol	46 (45)	18	17 (37)	1.2	(0.5–2.8)	0.61
Injecting	35 (29)	1	13 (37)	1.5	(0.7–3.4)	0.34
Using more than 3 ‘bags’ heroin per day	60 (53)	7	18 (30)	0.9	(0.4–2.0)	0.81
Pre-treatment UDS^b^ positives						
Benzodiazepines	71 (59)		26 (37)	1.6	(0.7–3.6)	0.25
Methadone	67 (56)		20 (30)	0.8	(0.4–1.6)	0.49
Cocaine	13 (11)		3 (23)	0.6	(0.2–2.3)	0.54^a^
Cannabis	47 (47)	20	13 (28)	0.7	(0.3–1.6)	0.38
Early Treatment						
Suboxone Commenced	32 (27)		7 (22)	0.5	(0.2–1.3)	0.13
At least one heroin negative UDS during induction	50 (46)	12	14 (28)	0.6	(0.3–1.4)	0.28

^a^*P* value calculated using Fishers Exact Test Statistic as estimated value in cell was less than 5

^b^UDS = Urine drug screen

^c^This column indicates the proportion (%) of the total group showing that characteristic (e.g. 51% of total group are female)

^d^This column indicates the proportion (%) of the subgroup with the characteristic who are still in treatment at month 12 (e.g. 25/61 [41%] of the females were still in treatment at month 12)

### Cessation of heroin use and other drugs

Table 2 presents the results of urine drug screens from the subgroup of patients who persisted with OST for at least 12 months. This table indicates the proportion demonstrating abstinence from each drug at each time period examined. During the third month of treatment 8 (21% [95% confidence interval (CI) = 9–36%]) were abstinent from heroin. Six (15% [95%CI = 6–31%]) were abstinent from heroin, cocaine and benzodiazepines. At month 12, there were 18 (46% [95%CI = 30–63%]) abstinent from heroin, of whom nine (23% [95%CI = 11–39%]) were also abstinent from cocaine and benzodiazepines.

**Table 2:** Evidence of drug use from urine drug screens (UDS), at baseline and during treatment, among 39 patients who persisted with treatment for 12 months

	Month 1				Month 3		Month 6		Month 12		Baseline vs Month 3	Baseline vs Month 12	Month 3 vs Month 6	Month 3 vs Month 12
	Pre-treatment baseline		Induction phase^b^											
Median number of UDS conducted (range)	3 (2–4)		4 (0–7)		7 (2–10)		7 (1–9)		8 (1–11)		Related samples Wilcoxon signed rank test *p* value			
Median % of UDS heroin negative (interquartile range)	0 (0–0)		0 (0–33)		50 (0–89)		40 (0–100)		87 (33–100)		< 0.001	< 0.001	0.86	0.01
All UDS negative during period											*p* value from McNemar test			
Heroin	1	(3)	4	(11)	8	(21)	12	(31)	18	(46)	0.04	< 0.001	0.34	0.04
Benzodiazepine	13	(33)	18	(50)	15	(38)	18	(46)	15	(38)	0.77	0.79	0.45	1.0
Cannabis^a^	19/32	(59)	18/28	(64)	20/37	(54)	16/33	(48)	14/36	(39)	0.69	0.11	1.0	0.29
Cocaine	36	(92)	36	(100)	32	(82)	31	(79)	33	(85)	0.22	0.45	1.0	1.0
Heroin, benzos & cocaine	0	(0)	1	(3)	6	(15)	8	(21)	9	(23)	n/a	n/a	0.63	0.55

^a^Urine drug screens were randomly tested for cannabis, which caused information on use to be missing for some patients during each period

^b^Three patients did not have any urine screens during induction

*n/a* not applicable

### Changes in drug use over time

Table 2 also indicates that heroin abstinence increased significantly from baseline to three months, from baseline to twelve months and from third month to twelfth month. Rates of use of other substances did not change significantly from baseline. When looking beyond heroin abstinence to examine changes in the proportions of negative urine screens, it also emerged that there were significant reductions in heroin use between baseline and month 3 and from month 3 to month 12. However, there was no significant change in heroin use between months 3 and 6. When comparing the proportions of heroin negative urine screens between months 3 and 12, we found that eight (21%) patients deteriorated, six (15%) were unchanged and 25 (64%) improved.

### Correlates of heroin abstinence

There were very few pre-treatment characteristics significantly associated with heroin abstinence at month 12. Full results of this analysis are presented in Additional file 1: Table S1 in an on-line supplement. None of the patients who had a previous psychiatric admission were abstinent (*p* = 0.02). Abstinence was not significantly associated with higher medication dose (*p* = 0.88). All of the patients who were using cocaine during month 12 were also using heroin (*p* = 0.02). Early reductions in heroin use, as evidenced by provision of at least one heroin negative sample during induction tended to be associated with reduced likelihood of heroin abstinence at month 12 (*p* = 0.07).

We repeated the analysis, with imputed positive heroin results for the 43 patients who had an unplanned discharge. This indicated that heroin abstinence was significantly associated with being in an intimate/sexual relationship with another heroin user (OR 3.4 [95%CI 1.1–10.0], *p* = 0.02). No other baseline characteristic was significantly associated with abstinence in that analysis with imputed results.

### Persistence with OST

**Fig. 1:**
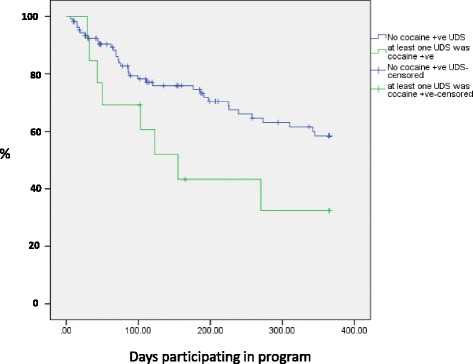
Unplanned treatment exit by 120 adolescents on OST; impact of cocaine use at treatment entry

Information on attrition during treatment is reported in Table 3. Overall, the estimated length of time to 25% leaving treatment in an unplanned manner was 120 (Standard Error 41) days. The table only presents results of the covariates where the Log Rank test indicated a *p* value of less than 0.2, although all covariates in Table 1 were explored. There was no significant difference between those commenced on Buprenorphine or methadone (log rank test statistic 1.04, *p* = 0.31). To further assist understanding of the analysis and the pattern of drop out, an example is provided in Fig. 1, which contrasts patients who showed UDS evidence of cocaine use during assessment against those without such cocaine use.

**Table 3:** Unplanned treatment exit among 120 heroin dependent adolescents during the first year on opioid substitution treatment (OST)

	Univariate analysis^a^				Multivariate cox regression		
	Estimated 25% drop out		Log rank statistic	*P* value	Hazard ratio (HR)	(95% CI of HR)	*P* value
	Days	(SE)					
Overall	120	(41)					
Socio-demographic characteristics							
Sex							
Female	75	(9)					
Male	225	(55)	2.36	0.13			
Age							
Under 18 years	120	(45)					
Aged 18 years	62	(69)	0.14	0.70			
Early school leaver							
Left Education under age 15	75	(18)					
Left school after 15th birthday	197	(69)	1.83	0.18			
Family of origin							
Has two parents	270				1.0		
Single parent/other relative/adopted	75	(19)	8.56	0.003	3.5	(1.7–6.9)	0.001
Own children							
Has a child	72	(61)			5.3	(1.9–14.8)	0.001
No children	123	(40)	5.11	0.02	1.0		
History of being in social care							
Has been in care	75	(28)					
Never in social care	155	(41)	3.52	0.06			
Parental heroin use							
No heroin use by parents	186	(66)					
A parent has heroin problems	72	(52)	2.91	0.09			
Heroin use by partner							
Partner has used heroin	270	(89)			1.0		
No partner/No heroin use by partner	86	(19)	3.53	0.06	2.1	(1.1–4.3)	0.03
Accommodation in past month							
Homeless or hostel	69	(25)					
Stable accommodation	176	(54)	2.40	0.12			
Deliberate self harm (DSH)							
Has a history of DSH	75	(28)			1.8	(1.0–3.3)	0.07
No history of past DSH	176	(67)	2.21	0.14	1.0		
Pre-treatment Drug Use							
Lifetime Cocaine							
Use	103	(29)					
No use	176	(69)	2.99	0.08			
Past month non-prescribed methadone use							
Self reported Use	197	(29)					
No self reported use	73	(23)	3.42	0.07			
Cocaine							
Positive baseline urine drug screen	50	(15)			3.2	(1.4–7.4)	0.006
All baseline urine drug screens negative	176	(55)	3.97	< 0.05	1.0		
Quantity of heroin use per day							
Using 3 ‘bags’ or more per day	69	(19)					
Using less than 3 ‘bags’ day	225	(40)	5.77	0.02			
Dose of OST at month three^b^							
Dose >50mgs methadone (or equivalent)	239	(24)					
Dose <= 50mgs methadone (or equivalent)	> 365		2.90	0.09			

^a^Kaplan Meier Survival analysis. The model estimate of the number of days to unplanned discharge by 25% of patients with that characteristic is reported, for comparison

^b^includes the 72 people being prescribed OST after 90 days, with methadone equivalent dose for those on buprenorphine being multiplied by 5 (i.e. if on 12mgs of buprenorphine, assigned value of 60mgs in methadone equivalents)

The Cox regression analysis (see Table 3) indicated that patients who had no children, grew up in families with two parents, were in an intimate relationship with another heroin user and were abstinent from cocaine in pre-treatment drug screens demonstrated significantly lower rates of unplanned exit from treatment.

Of the 20 people commenced on buprenorphine who persisted with treatment for three months, six (30%) had switched to methadone at that point. Only one person (2%) had switched from methadone to buprenorphine by month three. Seven people who commenced buprenorphine remained in treatment for 12 months, by which time six has switched to methadone. Only two of the 32 commenced on methadone had switched to buprenorphine by month 12.

## Discussion

### Reductions in heroin use and incremental change

Heroin use is associated with a vast array of risks [2, 5, 25]. A key goal of OST is to assist patients in reducing, and ideally stopping, their use of heroin [25]. Our study indicates that this treatment delivers substantial reductions in heroin use among adolescents. Almost half of these young patients who persisted with OST for a year demonstrated complete cessation of heroin use during their twelfth month of treatment. Previous studies of OST in adolescent heroin dependence have largely ignored the issue of abstinence during treatment. In a slightly older cohort, Kellogg et al. reported that patients who persisted with OST were heroin abstinent every other month on average, although urine drug screens were conducted inconsistently which increases the possibility that occasional use was not detected in that study [15].

The rate of heroin abstinence in month twelve constituted a significant improvement compared to the third month of treatment, at which point one in five demonstrated abstinence. This is consistent with one other study in adolescents and also with research on adult patients showing that longer treatment with OST builds incremental improvements in outcome [8, 14, 15, 26]. In their NIDA clinical trial, Woody et al. found a rapid return to heroin use once patients were tapered off their three months of buprenorphine treatment [9].

The confirmation that adolescents on OST can achieve substantial reductions in heroin use, with many doing so very early in treatment, should act as source of optimism for clinicians. It suggests that it is realistic for staff and treatment services to have a high expectation of reduced heroin use early in treatment and to be confident that many patients will achieve periods of sustained abstinence during treatment.

We found no indication of a relationship between medication dose and heroin abstinence. There was no upper ceiling dose used in this service and our finding may indicate that dose was appropriately titrated against clinical need. There is research to indicate increased heroin use at lower OST doses in both adolescents and adults [18, 27]. In contrast, Kellogg et al. detected greater heroin use among young adults on higher methadone doses [15].

### Treatment retention

The second main focus of this study was treatment retention. We found that one in four patients had an unplanned treatment exit by 120 days. Retention rates were similar in DARP in the 1970s and in the more recent study by Kellogg et al. [8, 15]. Those two studies examined methadone treatment. A number of studies examining use of buprenorphine in other settings report much higher rates of attrition among adolescent patients [16–18]. In contrast to Bell & Mutch, we found no significant difference between the two opioid agonist medications, although many patients in our study did switch from buprenorphine to methadone during treatment [16]. One factor which complicates comparisons of retention rates across all of these studies relates to the variable criteria used to define ‘drop out’. In our study, patients who recommenced regular attendance after a period of up to four weeks non-attendance were viewed as still being in their index treatment episode. Other studies have viewed patients as having dropped out with much shorter periods of non-attendance.

### Use of other substances

Unfortunately, half of those who were heroin abstinent demonstrated some ongoing use of benzodiazepines. Overall, the rates of abstinence from drugs other than heroin did not increase significantly from pre-treatment levels. Studies of OST in adults suggest that they can achieve reductions in use of other drugs [14, 26]. However, previous research on younger cohorts has also failed to demonstrate reductions in use of other drugs and the DARP study found some evidence of increased use of cannabis and alcohol at follow-up [8, 15].

At this service, efforts were made to address cocaine use. Where use occurred it was discussed in counselling sessions and also during key working interventions. Our failure to successfully reduce cocaine use appears important for this patient group. Although cocaine use was not extensive among these heroin dependent adolescents, it seems to act as a negative prognostic factor for those who use it. Cocaine use at baseline was a significant predictor of treatment drop out. Cocaine use during OST was correlated with ongoing heroin use. A correlation between persistent heroin use and cocaine use has also been demonstrated by others in both adolescents and adults [15, 27, 28]. The adverse association between cocaine use and poorer treatment adherence was also reported by Kellogg et al. [15]. Adult studies of OST have made similar observations [29, 30]. For these reasons, efforts have been made in Ireland to implement interventions targeting cocaine use among adults attending OST. The results have been quite disappointing to date, with poor uptake of counselling treatments being a key obstacle [31]. With polydrug using adolescents, it has been shown in Ireland and elsewhere that their motivation to address their main drug may be very different to their motivation to address substances which they perceive as quite secondary [13]. In spite of these challenges, services working with heroin dependent adolescents should have protocols in place to assess and respond to use of cocaine and other substances.

### Association of systemic factors with outcome

Other potential prognostic factors were identified in the exploratory analysis, many of which suggested the importance of the family and social support system around the adolescent. Some characteristics of family systems may provide support to an adolescent who is facing a severe drug use disorder while other family types may add to the strain on an adolescent who is already coping poorly [32, 33]. Our finding of increased abstinence when analysis included imputed data, and of better treatment retention, in those with a heroin using sexual partner was unexpected. The general view in addiction treatment is that increased social ties with other drug users brings poorer outcome [33]. Interestingly, in a separate study examining outcome after inpatient detoxification of heroin dependent adults in Ireland, it also emerged that being in a relationship with another heroin user was associated with better outcome [34]. Although these findings are counter-intuitive, the issue may warrant further exploration by other researchers. We have previously reported that female patients are much more likely to be in an intimate relationship with another heroin user [13]. Typically partners enter treatment at the same time. It seems possible that a shared goal and a shared understanding of the challenges faced by a partner may support rather than undermine recovery [35].

We found that adolescents from families with two parents had better retention. Crome et al. also found better outcome with increased family support [36]. Involvement of parents in both assessment and treatment was prioritised by the service [21]. Efforts were made to actively include parents in the care plan. Family involvement in adolescent addiction treatment has been shown to improve outcomes [37]. It may be the case that families which include two parents are better able to cope with the strain which addiction places upon the family system, and therefore better positioned to provide support to their son or daughter [32].

A final systemic factor which was correlated with outcome was being a young parent. While only a small number of these adolescent patients had a child themselves, those who were faced with this additional strain on their personal resources were less likely to persist with treatment. Drop out occurred in spite of the efforts by the service to support these young parents via provision of crèche facilities on site. Others have highlighted the particular challenges faced by single parents with addiction issues, and some services have developed targeted programs of support such as the Parents under Pressure intervention [38].

Those who had a previous psychiatric admission appeared less likely to be abstinent. Crome et al. also reported poorer outcome in those with a psychiatric history [36]. However, Subramaniam et al. found that greater baseline psychiatric problems was associated with better outcome in the NIDA clinical trial of buprenorphine [10]. Our previous research has indicated that there are improvements in mental health symptoms among the adolescents attending OST at this service and lower depressive symptoms correlate with reduced heroin use [21].

### Strengths and limitations

The strengths of this study include the sample size which is quite large relative to other studies of OST in this age range. However, the sample size is still relatively small for statistical purposes, with limited power. In view of the large number of statistical tests conducted in this study, and the decision not to alter the *p* value via a Bonferoni correction, this raises the possibility that some significant findings may also constitute type 1 statistical errors.

The treatment intervention was delivered at a single site making it easier for clinicians elsewhere to determine how applicable our findings may be to their own treatment setting. The monitoring of drug use by twice weekly supervised UDS constitutes a very thorough level of evaluation of drug use and we can be reasonably confident that the patients whom we determined to be abstinent are truly abstinent. A limitation can arise from assessing change via very regular urine screens. For example, a patient could change from injecting heroin four times a day at treatment entry to smoking heroin just twice a week. While this constitutes a very positive achievement from a harm reduction perspective, all UDS would be likely to remain opiate positive and our results would indicate no change in such a scenario.

## Conclusions

This study adds to the currently limited evidence base for OST in adolescents who are heroin dependent. The positive outcomes appear broadly similar to those achieved in adults, among whom there is widespread acceptance of this treatment. One of the lingering doubts about OST in adolescents since the DARP study relates to the limited success in achieving reductions in use of other drugs [8]. In spite of our efforts to address this use, we also found that levels of use of other substances remained stubbornly elevated. This does not negate the fact that the reductions in heroin use were substantial. Importantly, abstinence increased significantly from month three to one year, with half of the patient group on OST having ceased heroin use during their full twelfth month of treatment. It seems difficult to predict which patients are going to persist with this treatment and to have better outcomes. However, use of cocaine, both prior to and during treatment, appears to be a negative prognostic factor. With adolescent patients it appears to be particularly important to be mindful of potential systemic factors which may either support or undermine treatment success.

Overall, the efforts currently being made to ensure greater provision of OST to adolescents by groups such as the American Academy of Pediatrics appear appropriate and timely [2]. Also, where treatment is commenced, we echo the view of Woody et al. that “clinicians should be in no hurry to stop an effective medication simply because the patient is young” [9].

## Additional file

Additional file 1: **Table S1.** Heroin abstinence during month 12 among 39 heroin dependent adolescents on opioid agonist treatment. (DOCX 19 kb)

## Abbreviations

BT: Buprenorphine treatment
CI: Confidence interval
DARP: Drug abuse reporting program
EDDP: 5-ethylidene-1,5-dimethyl-3, 3-diphenylpyrrolidine
MT: Methadone treatment
NDTC: National Drug Treatment Centre
NIDA: National Institute of Drug Abuse
OST: Opioid substitution treatment
UDS: Urine drugs screens
YPP: Young Persons’ Programme
