# Supplementary material for: Opioid substitution treatment and heroin dependent adolescents: reductions in heroin use and treatment retention over twelve months
Source: BMC Pediatr. 2018 May 4;18:151. doi: 10.1186/s12887-018-1137-4 (PMC5936020; doi:10.1186/s12887-018-1137-4)
Supplement: Supplementary file 1 — Table S1. Heroin abstinence during month 12 among 39 heroin dependent adolescents on opioid agonist treatment. (DOCX 19 kb) [file 12887_2018_1137_MOESM1_ESM.docx]

**Table S1.** Heroin abstinence during month 12 among 39 heroin dependent adolescents on opioid agonist treatment

|  |  |  | **Total** | **Heroin Abstinent during month 12** | | | |
| --- | --- | --- | --- | --- | --- | --- | --- |
|  |  |  | N (%) | N (%) | OR | 95% CI OR | P value |
|  |  |  |  |  |  |  |  |
| ***Number in Treatment*** | | | 39 (100) | 18 (46) |  |  |  |
|  | | |  |  |  |  |  |
| ***Socio-demographic characteristics*** | | |  |  |  |  |  |
|  | Female | | 25 (64) | 13 (52) | 2.0 | (0.5-7.5) | 0.33 |
|  | Aged under 18.0 years | | 35 (90) | 16 (46) | 0.8 | (0.1-6.7) | 1.0^ |
|  | Left school under 15 years | | 18 (47) | 9 (50) | 1.2 | (0.3-4.4) | 0.76 |
|  | Not in employment, education or training | | 29 (76) | 14 (48) | 1.2 | (0.3-5.2) | 1.0^ |
|  | Two parent family support | | 27 (71) | 11 (40) | 0.6 | (0.1-2.4) | 0.49 |
|  | Has a child | | 0 (0) | N/a |  |  |  |
|  | Has been in care | | 10 (27) | 6 (60) | 2.6 | (0.6-11.3) | 0.27^ |
|  | Sibling heroin Use | | 17 (45) | 7 (41) | 0.8 | (0.2-2.8) | 0.69 |
|  | Parental heroin Use | | 6 (16) | 3 (50) | 1.3 | (0.2-7.4) | 1.0^ |
|  | Partner uses heroin | | 18 (47) | 11 (61) | 2.9 | (0.8-10.9) | 0.11 |
|  | Homeless or hostel in past month | | 11 (28) | 5 (46) | 1.0 | (0.2-3.9) | 0.96 |
|  | Previous criminal convictions | | 14 (38) | 5 (36) | 0.6 | (0.2-2.4) | 0.47 |
|  | Ever incarcerated | | 8 (22) | 2 (25) | 0.4 | (0.1-1.8) | 0.24 |
|  | | |  |  |  |  |  |
| ***Psychiatric History*** | | |  |  |  |  |  |
|  | Ever seen a psychiatrist | | 22 (56) | 9 (41) | 0.6 | (0.2-2.2) | 0.46 |
|  | Inpatient psychiatric admission | | 6 (15) | 0 (0) |  |  | 0.02^ |
|  | Past DSH | | 12 (33) | 4 (33) | 0.4 | (0.1-1.8) | 0.24 |
|  |  | |  |  |  |  |  |
| ***Substance Use*** | | |  |  |  |  |  |
|  | *Lifetime Drug Use* | |  |  |  |  |  |
|  |  | Non-prescribed benzodiazepines | 36 (92) | 17 (47) | 1.8 | (0.1-21.5) | 1.0 |
|  |  | Non-prescribed methadone | 30 (77) | 14 (47) | 1.1 | (0.3-5.1) | 0.71 |
|  |  | Cocaine | 30 (83) | 12 (40) | 0.1 | (0.01-1.3) | 0.08 |
|  |  | Injected | 17 (44) | 8 (47) | 1.1 | (0.3-3.8) | 0.92 |
|  |  | Commenced heroin under 15 years of age | 20 (51) | 10 (50) | 1.4 | (0.4-4.9) | 0.62 |
|  |  | Regular heroin use for more at least 12 months | 26 (67) | 11 (42) | 0.6 | (0.2-2.4) | 0.50 |
|  |  | |  |  |  |  |  |
|  | *Past Month Drug Use* | |  |  |  |  |  |
|  |  | Non-prescribed benzodiazepines | 26 (67) | 12 (46) | 1.0 | (0.3-3.8) | 1.0^ |
|  |  | Non-prescribed methadone | 27 (69) | 13 (48) | 1.3 | (0.3-5.1) | 0.71 |
|  |  | Cocaine | 10 (26) | 4 (40) | 0.8 | (0.2-3.3) | 1.0^ |
|  |  | Cannabis | 27 (80) | 12 (44) | 0.3 | (0.1-1.9) | 0.40^ |
|  |  | Amphetamine | 4 (13) | 1 (25) | 0.3 | (0.03-3.1) | 0.60^ |
|  |  | Alcohol | 17 (50) | 6 (35) | 0.4 | (0.1-1.5) | 0.17 |
|  |  | Injecting | 13 (33) | 5 (38) | 0.6 | (0.2-2.4) | 0.50 |
|  |  | Using more than 3 ‘bags’ heroin per day | 18 (51) | 4 (44) | 0.7 | (0.2-2.7) | 0.62 |
|  |  |  |  |  |  |  |  |
|  | *Pre-treatment urine drug screen (UDS)*^~^  *positives* | |  |  |  |  |  |
|  |  | Benzodiazepines | 26 (67) | 14 (54) | 2.6 | (0.6-10.7) | 0.17 |
|  |  | Methadone | 20 (51) | 10 (50) | 1.4 | (0.4-4.9) | 0.62 |
|  |  | Cocaine | 3 (8) | 0 (0) |  |  | 0.24 |
|  |  | Cannabis | 13 (41) | 6 (46) | 0.6 | (0.2-2.6) | 0.51 |
|  |  |  |  |  |  |  |  |
| ***Treatment*** | | |  |  |  |  |  |
|  |  | Suboxone Commenced | 7 (18) | 4 (57) | 1.7 | (0.3-8.9) | 0.68 |
|  |  | At least one opiate negative UDS during induction | 14 (39) | 4 (29) | 0.3 | (0.1-1.2) | 0.07 |
|  |  | Heroin abstinent throughout month 3 | 8 (21) | 3 (38) | 0.6 | (0.1-3.2) | 0.70 |
|  |  | Heroin abstinent throughout month 6 | 12 (31) | 8 (67) | 3.4 | (0.8-14.3) | 0.09 |
|  |  | Methadone dose at month 12 >50mgs ^#^ | 20 (51) | 9 (45) | 0.9 | (0.3-3.2) | 0.88 |
|  |  | UDS at month 12 indicate Benzodiazepine use | 24 62) | 9 (38) | 0.4 | (0.1-1.5) | 0.17 |
|  |  | UDS at month 12 indicate cocaine use | 6 (15) | 0 (0) |  |  | 0.02^ |
|  |  | UDS at month 12 indicate cannabis use | 22 (58) | 10 (46) | 0.8 | (0.2-3.0) | 0.78 |
|  |  | |  |  |  |  |  |

^^^ P value calculated using Fishers Exact Test Statistic as estimated value in cell was less than 5

^#^ For those patients on buprenorphine, dose is multiplied by 5 to approximate to equivalent methadone dose

^~^ UDS = Urine drug screens, which were conducted twice weekly on average.

^a^ This column indicates the proportion (%) of the total group in treatment at month 12 showing that characteristic (e.g. 25/39 [64%] of those in treatment at month 12 were female)

^b^ This column indicates the proportion (%) of the subgroup with the characteristic who are heroin abstinent at month 12 (e.g. 13/25 [52%] of the females who were still in treatment at month 12 were heroin abstinent during that month )
